# Supplementary material for: Ferulic Acid reduces amyloid beta mediated neuroinflammation through modulation of Nurr1 expression in microglial cells
Source: PLoS One. 2023 Aug 17;18(8):e0290249. doi: 10.1371/journal.pone.0290249 (PMC10434858; doi:10.1371/journal.pone.0290249)
Supplement: S1 Dataset — (PDF) [file pone.0290249.s001.pdf]

Figure 2 B:

Tukey's multiple comparisons test One Way ANOVA	Mean Diff.	95.00% CI of diff.	Below threshold?	Summary	Adjusted P Value
Ctrl vs. 50	-1.585	-2.398 to -0.7716	Yes	****	<0.0001
Ctrl vs. 100	-0.4790	-1.292 to 0.3344	No	ns	0.4603
Ctrl vs. 250	0.8940	0.08055 to 1.707	Yes	*	0.0247
Ctrl vs. 500	2.423	1.610 to 3.236	Yes	****	<0.0001
50 vs. 100	1.106	0.2926 to 1.919	Yes	**	0.0031
50 vs. 250	2.479	1.666 to 3.292	Yes	****	<0.0001
50 vs. 500	4.008	3.195 to 4.821	Yes	****	<0.0001
100 vs. 250	1.373	0.5596 to 2.186	Yes	***	0.0002
100 vs. 500	2.902	2.089 to 3.715	Yes	****	<0.0001
250 vs. 500	1.529	0.7156 to 2.342	Yes	****	<0.0001

Test details	Mean 1	Mean 2	Mean Diff.	SE of diff.	n1	n2
Ctrl vs. 50	3.223	4.808	-1.585	0.2863	10	10
Ctrl vs. 100	3.223	3.702	-0.4790	0.2863	10	10
Ctrl vs. 250	3.223	2.329	0.8940	0.2863	10	10
Ctrl vs. 500	3.223	0.8000	2.423	0.2863	10	10
50 vs. 100	4.808	3.702	1.106	0.2863	10	10
50 vs. 250	4.808	2.329	2.479	0.2863	10	10
50 vs. 500	4.808	0.8000	4.008	0.2863	10	10
100 vs. 250	3.702	2.329	1.373	0.2863	10	10
100 vs. 500	3.702	0.8000	2.902	0.2863	10	10
250 vs. 500	2.329	0.8000	1.529	0.2863	10	10

Figure 2 C:

a : NURR1

Tukey's multiple comparisons test One Way ANOVA	Mean Diff.	95.00% CI of diff.	Below threshold?	Summary	Adjusted P Value
0 (Ctrl) vs. 50	-5.290	-6.429 to -4.151	Yes	****	<0.0001
0 (Ctrl) vs. 100	-5.083	-6.223 to -3.944	Yes	****	<0.0001
0 (Ctrl) vs. 250	-3.193	-4.333 to -2.054	Yes	****	<0.0001
0 (Ctrl) vs. 500	-0.3300	-1.469 to 0.8093	No	ns	0.8695
50 vs. 100	0.2067	-0.9326 to 1.346	No	ns	0.9724
50 vs. 250	2.097	0.9574 to 3.236	Yes	***	0.0009
50 vs. 500	4.960	3.821 to 6.099	Yes	****	<0.0001
100 vs. 250	1.890	0.7507 to 3.029	Yes	**	0.0020
100 vs. 500	4.753	3.614 to 5.893	Yes	****	<0.0001
250 vs. 500	2.863	1.724 to 4.003	Yes	****	<0.0001

Test details	Mean 1	Mean 2	Mean Diff.	SE of diff.	n1	n2	q	DF
0 (Ctrl) vs. 50	0.9800	6.270	-5.290	0.3462	3	3	21.61	10
0 (Ctrl) vs. 100	0.9800	6.063	-5.083	0.3462	3	3	20.77	10
0 (Ctrl) vs. 250	0.9800	4.173	-3.193	0.3462	3	3	13.05	10
0 (Ctrl) vs. 500	0.9800	1.310	-0.3300	0.3462	3	3	1.348	10
50 vs. 100	6.270	6.063	0.2067	0.3462	3	3	0.8443	10
50 vs. 250	6.270	4.173	2.097	0.3462	3	3	8.565	10
50 vs. 500	6.270	1.310	4.960	0.3462	3	3	20.26	10
100 vs. 250	6.063	4.173	1.890	0.3462	3	3	7.721	10
100 vs. 500	6.063	1.310	4.753	0.3462	3	3	19.42	10
250 vs. 500	4.173	1.310	2.863	0.3462	3	3	11.70	10

b : IL1β

Tukey's multiple comparisons test One Way ANOVA	Mean Diff.	95.00% CI of diff.	Below threshold?	Summary	Adjusted P Value
0 (Ctrl) vs. 50	0.4067	0.2833 to 0.5300	Yes	****	<0.0001
0 (Ctrl) vs. 100	0.3167	0.1933 to 0.4400	Yes	****	<0.0001
0 (Ctrl) vs. 250	0.1867	0.06333 to 0.3100	Yes	**	0.0039
0 (Ctrl) vs. 500	-0.7767	-0.9000 to -0.6533	Yes	****	<0.0001
50 vs. 100	-0.09000	-0.2133 to 0.03334	No	ns	0.1919
50 vs. 250	-0.2200	-0.3433 to -0.09666	Yes	**	0.0011
50 vs. 500	-1.183	-1.307 to -1.060	Yes	****	<0.0001
100 vs. 250	-0.1300	-0.2533 to -0.006664	Yes	*	0.0379
100 vs. 500	-1.093	-1.217 to -0.9700	Yes	****	<0.0001
250 vs. 500	-0.9633	-1.087 to -0.8400	Yes	****	<0.0001

Test details	Mean 1	Mean 2	Mean Diff.	SE of diff.	n1	n2	q	DF
0 (Ctrl) vs. 50	0.9633	0.5567	0.4067	0.03748	3	3	15.35	10
0 (Ctrl) vs. 100	0.9633	0.6467	0.3167	0.03748	3	3	11.95	10
0 (Ctrl) vs. 250	0.9633	0.7767	0.1867	0.03748	3	3	7.044	10
0 (Ctrl) vs. 500	0.9633	1.740	-0.7767	0.03748	3	3	29.31	10
50 vs. 100	0.5567	0.6467	-0.09000	0.03748	3	3	3.396	10
50 vs. 250	0.5567	0.7767	-0.2200	0.03748	3	3	8.302	10
50 vs. 500	0.5567	1.740	-1.183	0.03748	3	3	44.65	10
100 vs. 250	0.6467	0.7767	-0.1300	0.03748	3	3	4.906	10
100 vs. 500	0.6467	1.740	-1.093	0.03748	3	3	41.26	10
250 vs. 500	0.7767	1.740	-0.9633	0.03748	3	3	36.35	10

C : IL10

Tukey's multiple comparisons test One Way ANOVA	Mean Diff.	95.00% CI of diff.	Below threshold?	Summary	Adjusted P Value
0 (Ctrl) vs. 50	-1.463	-1.679 to -1.248	Yes	****	<0.0001
0 (Ctrl) vs. 100	-0.9367	-1.152 to -0.7212	Yes	****	<0.0001
0 (Ctrl) vs. 250	-0.4667	-0.6821 to -0.2512	Yes	***	0.0002
0 (Ctrl) vs. 500	0.2067	-0.008753 to 0.4221	No	ns	0.0615
50 vs. 100	0.5267	0.3112 to 0.7421	Yes	****	<0.0001
50 vs. 250	0.9967	0.7812 to 1.212	Yes	****	<0.0001
50 vs. 500	1.670	1.455 to 1.885	Yes	****	<0.0001
100 vs. 250	0.4700	0.2546 to 0.6854	Yes	***	0.0002
100 vs. 500	1.143	0.9279 to 1.359	Yes	****	<0.0001
250 vs. 500	0.6733	0.4579 to 0.8888	Yes	****	<0.0001

Test details	Mean 1	Mean 2	Mean Diff.	SE of diff.	n1	n2	q	DF
0 (Ctrl) vs. 50	0.9700	2.433	-1.463	0.06546	3	3	31.62	10
0 (Ctrl) vs. 100	0.9700	1.907	-0.9367	0.06546	3	3	20.24	10
0 (Ctrl) vs. 250	0.9700	1.437	-0.4667	0.06546	3	3	10.08	10
0 (Ctrl) vs. 500	0.9700	0.7633	0.2067	0.06546	3	3	4.465	10
50 vs. 100	2.433	1.907	0.5267	0.06546	3	3	11.38	10
50 vs. 250	2.433	1.437	0.9967	0.06546	3	3	21.53	10
50 vs. 500	2.433	0.7633	1.670	0.06546	3	3	36.08	10
100 vs. 250	1.907	1.437	0.4700	0.06546	3	3	10.15	10
100 vs. 500	1.907	0.7633	1.143	0.06546	3	3	24.70	10
250 vs. 500	1.437	0.7633	0.6733	0.06546	3	3	14.55	10

Figure 3 :

3 B :

Tukey's multiple comparisons test One Way ANOVA	Mean Diff.	95.00% CI of diff.	Below threshold?	Summary	Adjusted P Value
Ctrl vs. Aß	2.423	1.635 to 3.211	Yes	****	<0.0001
Ctrl vs. FA	-1.585	-2.373 to -0.7973	Yes	****	<0.0001
Ctrl vs. Aß+FA	0.1080	-0.6797 to 0.8957	No	ns	0.9825
Aß vs. FA	-4.008	-4.796 to -3.220	Yes	****	<0.0001
Aß vs. Aß+FA	-2.315	-3.103 to -1.527	Yes	****	<0.0001
FA vs. Aß+FA	1.693	0.9053 to 2.481	Yes	****	<0.0001

Test details	Mean 1	Mean 2	Mean Diff.	SE of diff.	n1	n2	q	DF
Ctrl vs. Aß	3.223	0.8000	2.423	0.2925	10	10	11.72	36
Ctrl vs. FA	3.223	4.808	-1.585	0.2925	10	10	7.664	36
Ctrl vs. Aß+FA	3.223	3.115	0.1080	0.2925	10	10	0.5222	36
Aß vs. FA	0.8000	4.808	-4.008	0.2925	10	10	19.38	36
Aß vs. Aß+FA	0.8000	3.115	-2.315	0.2925	10	10	11.19	36
FA vs. Aß+FA	4.808	3.115	1.693	0.2925	10	10	8.186	36

3 C :

Tukey's multiple comparisons Test<br>One Way ANOVA	Mean Diff.	95.00% CI of diff.	Below threshold?	Summary	Adjusted P Value
Amoeboid					
Ctrl vs. Aß	-17.60	-23.27 to -11.93	Yes	****	<0.0001
Ctrl vs. FA	13.20	7.532 to 18.87	Yes	****	<0.0001
Ctrl vs. Aß+FA	2.700	-2.968 to 8.368	No	ns	0.6010
Aß vs. FA	30.80	25.13 to 36.47	Yes	****	<0.0001
Aß vs. Aß+FA	20.30	14.63 to 25.97	Yes	****	<0.0001
FA vs. Aß+FA	-10.50	-16.17 to -4.832	Yes	****	<0.0001
Rod-like					
Ctrl vs. Aß	-7.600	-13.27 to -1.932	Yes	**	0.0037
Ctrl vs. FA	7.300	1.632 to 12.97	Yes	**	0.0058
Ctrl vs. Aß+FA	2.600	-3.068 to 8.268	No	ns	0.6301
Aß vs. FA	14.90	9.232 to 20.57	Yes	****	<0.0001
Aß vs. Aß+FA	10.20	4.532 to 15.87	Yes	****	<0.0001
FA vs. Aß+FA	-4.700	-10.37 to 0.9681	No	ns	0.1401
Ramified					
Ctrl vs. Aß	25.20	19.53 to 30.87	Yes	****	<0.0001
Ctrl vs. FA	-21.90	-27.57 to -16.23	Yes	****	<0.0001
Ctrl vs. Aß+FA	-9.100	-14.77 to -3.432	Yes	***	0.0003
Aß vs. FA	-47.10	-52.77 to -41.43	Yes	****	<0.0001
Aß vs. Aß+FA	-34.30	-39.97 to -28.63	Yes	****	<0.0001
FA vs. Aß+FA	12.80	7.132 to 18.47	Yes	****	<0.0001

Test details	Mean 1	Mean 2	Mean Diff.	SE of diff.	N1	N2	q	DF
Amoeboid								

Ctrl vs. Aß	26.80	44.40	-17.60	2.172	10	10	11.46	108.0
Ctrl vs. FA	26.80	13.60	13.20	2.172	10	10	8.594	108.0
Ctrl vs. Aß+FA	26.80	24.10	2.700	2.172	10	10	1.758	108.0
Aß vs. FA	44.40	13.60	30.80	2.172	10	10	20.05	108.0
Aß vs. Aß+FA	44.40	24.10	20.30	2.172	10	10	13.22	108.0
FA vs. Aß+FA	13.60	24.10	-10.50	2.172	10	10	6.836	108.0
Rod-like								
Ctrl vs. Aß	36.10	43.70	-7.600	2.172	10	10	4.948	108.0
Ctrl vs. FA	36.10	28.80	7.300	2.172	10	10	4.753	108.0
Ctrl vs. Aß+FA	36.10	33.50	2.600	2.172	10	10	1.693	108.0
Aß vs. FA	43.70	28.80	14.90	2.172	10	10	9.701	108.0
Aß vs. Aß+FA	43.70	33.50	10.20	2.172	10	10	6.641	108.0
FA vs. Aß+FA	28.80	33.50	-4.700	2.172	10	10	3.060	108.0
Ramified								
Ctrl vs. Aß	35.60	10.40	25.20	2.172	10	10	16.41	108.0
Ctrl vs. FA	35.60	57.50	-21.90	2.172	10	10	14.26	108.0
Ctrl vs. Aß+FA	35.60	44.70	-9.100	2.172	10	10	5.925	108.0
Aß vs. FA	10.40	57.50	-47.10	2.172	10	10	30.67	108.0
Aß vs. Aß+FA	10.40	44.70	-34.30	2.172	10	10	22.33	108.0
FA vs. Aß+FA	57.50	44.70	12.80	2.172	10	10	8.334	108.0

3 D :

NURR1

Tukey's multiple comparisons test One Way ANOVA	Mean Diff.	95.00% CI of diff.	Below threshold?	Summary	Adjusted P Value
Ctrl vs. Aß	0.5600	0.1865 to 0.9335	Yes	**	0.0059
Ctrl vs. FA	-5.063	-5.437 to -4.690	Yes	****	<0.0001
Ctrl vs. Aß+FA	-1.460	-1.834 to -1.086	Yes	****	<0.0001
Aß vs. FA	-5.623	-5.997 to -5.250	Yes	****	<0.0001
Aß vs. Aß+FA	-2.020	-2.394 to -1.646	Yes	****	<0.0001
FA vs. Aß+FA	3.603	3.230 to 3.977	Yes	****	<0.0001

Test details	Mean 1	Mean 2	Mean Diff.	SE of diff.	n1	n2	q	DF
Ctrl vs. Aß	1.000	0.4400	0.5600	0.1166	3	3	6.790	8
Ctrl vs. FA	1.000	6.063	-5.063	0.1166	3	3	61.39	8
Ctrl vs. Aß+FA	1.000	2.460	-1.460	0.1166	3	3	17.70	8
Aß vs. FA	0.4400	6.063	-5.623	0.1166	3	3	68.18	8
Aß vs. Aß+FA	0.4400	2.460	-2.020	0.1166	3	3	24.49	8
FA vs. Aß+FA	6.063	2.460	3.603	0.1166	3	3	43.69	8

IL1β

Tukey's multiple comparisons test One Way ANOVA	Mean Diff.	95.00% CI of diff.	Below threshold?	Summary	Adjusted P Value
Ctrl vs. Aß	-2.540	-3.173 to -1.907	Yes	****	<0.0001
Ctrl vs. FA	0.6200	-0.01282 to 1.253	No	ns	0.0548
Ctrl vs. Aß+FA	-0.3500	-0.9828 to 0.2828	No	ns	0.3518
Aß vs. FA	3.160	2.527 to 3.793	Yes	****	<0.0001
Aß vs. Aß+FA	2.190	1.557 to 2.823	Yes	****	<0.0001
FA vs. Aß+FA	-0.9700	-1.603 to -0.3372	Yes	**	0.0052

Test details	Mean 1	Mean 2	Mean Diff.	SE of diff.	n1	n2	q	DF
Ctrl vs. Aß	1.000	3.540	-2.540	0.1976	3	3	18.18	8
Ctrl vs. FA	1.000	0.3800	0.6200	0.1976	3	3	4.437	8
Ctrl vs. Aß+FA	1.000	1.350	-0.3500	0.1976	3	3	2.505	8
Aß vs. FA	3.540	0.3800	3.160	0.1976	3	3	22.61	8
Aß vs. Aß+FA	3.540	1.350	2.190	0.1976	3	3	15.67	8
FA vs. Aß+FA	0.3800	1.350	-0.9700	0.1976	3	3	6.942	8

IL10

Tukey's multiple comparisons test One Way ANOVA	Mean Diff.	95.00% CI of diff.	Below threshold?	Summary	Adjusted P Value
Ctrl vs. Aß	0.6233	0.05784 to 1.189	Yes	*	0.0316
Ctrl vs. FA	-2.477	-3.042 to -1.911	Yes	****	<0.0001
Ctrl vs. Aß+FA	-1.557	-2.122 to -0.9912	Yes	****	<0.0001
Aß vs. FA	-3.100	-3.665 to -2.535	Yes	****	<0.0001
Aß vs. Aß+FA	-2.180	-2.745 to -1.615	Yes	****	<0.0001
FA vs. Aß+FA	0.9200	0.3545 to 1.485	Yes	**	0.0036

Test details	Mean 1	Mean 2	Mean Diff.	SE of diff.	n1	n2	q	DF
Ctrl vs. Aß	1.000	0.3767	0.6233	0.1766	3	3	4.992	8
Ctrl vs. FA	1.000	3.477	-2.477	0.1766	3	3	19.83	8
Ctrl vs. Aß+FA	1.000	2.557	-1.557	0.1766	3	3	12.47	8
Aß vs. FA	0.3767	3.477	-3.100	0.1766	3	3	24.83	8
Aß vs. Aß+FA	0.3767	2.557	-2.180	0.1766	3	3	17.46	8
FA vs. Aß+FA	3.477	2.557	0.9200	0.1766	3	3	7.368	8

Figure 4 B :

Tukey's multiple comparisons test One Way ANOVA	Mean Diff.	95.00% CI of diff.	Below threshold?	Summary	Adjusted P Value
Ctrl vs. Aß	6.700	3.432 to 9.968	Yes	****	<0.0001
Ctrl vs. FA	-20.10	-23.37 to -16.83	Yes	****	<0.0001
Ctrl vs. Aß+FA	-4.300	-7.568 to -1.032	Yes	**	0.0059
Aß vs. FA	-26.80	-30.07 to -23.53	Yes	****	<0.0001
Aß vs. Aß+FA	-11.00	-14.27 to -7.732	Yes	****	<0.0001
FA vs. Aß+FA	15.80	12.53 to 19.07	Yes	****	<0.0001

Test details	Mean 1	Mean 2	Mean Diff.	SE of diff.	n1	n2	q	DF
Ctrl vs. Aß	9.400	2.700	6.700	1.214	10	10	7.808	36
Ctrl vs. FA	9.400	29.50	-20.10	1.214	10	10	23.42	36
Ctrl vs. Aß+FA	9.400	13.70	-4.300	1.214	10	10	5.011	36
Aß vs. FA	2.700	29.50	-26.80	1.214	10	10	31.23	36
Aß vs. Aß+FA	2.700	13.70	-11.00	1.214	10	10	12.82	36
FA vs. Aß+FA	29.50	13.70	15.80	1.214	10	10	18.41	36
