# Supplementary material for: Ferulic Acid reduces amyloid beta mediated neuroinflammation through modulation of Nurr1 expression in microglial cells
Source: PLoS One. 2023 Aug 17;18(8):e0290249. doi: 10.1371/journal.pone.0290249 (PMC10434858; doi:10.1371/journal.pone.0290249)
Supplement: Attachment — Submitted filename: Response to Reviewers R2.docx [file pone.0290249.s003.docx]

**Dear Editor**

Thank you very much for comments we have checked all the citations and there is not any type of retracted citation in our paper. We double checked the references and citations according to the reviewer's comments. Also in the first revision we made a mistake in citations of one of the paragraphs and in this second revision we inserted lost citations.

Regards

Our answers to the comments of reviewers are as below.

**Dear reviewers**

Thank you very much for your detailed and advantageous comments which will definitely add value to our article. Below is the list of comments and amendments made for each of them in order. Also, a track changed version has been uploaded after applying the changes to the article.

Regards

**Comments and Answers:**

**Reviewer #1:**

**No Comment**

**Reviewer #2:**

**Comment 1:** The authors need to double check the citations. Various phrases still missing references and some need to double check the accuracy. For example, line 80-89. In the original submission the author mentioned the reference then they deleted in the revision version!!!

**Answer:** In the first revision we made a mistake in citations of one of the revised paragraphs and in this second revision we inserted lost citations in that paragraph lines 80-89. Also we added some citations in lines 293-296.

80-89: One of the transcription factors that can inhibit inflammation caused by Aβ is Nurr1 [26, 27], whose structural defects or reduced expression have been shown to be associated with increased amyloid plaque deposition [26]. As a transcription factor, Nurr1 plays a crucial role in the development of dopaminergic neurons and contributes to the activation of neuroprotective and anti-inflammatory pathways [28]. Dysfunction or reduced expression of Nurr1 has been associated with impaired energy metabolism, mitochondrial dysfunction, and oxidative stress [29, 30]. Given the association between reduced Nurr1 expression and these changes in dopaminergic neurons, beside anti-synucleinopathy therapy, increasing Nurr1 expression has been suggested as a promising therapeutic target for Parkinson disease [28]. However, fewer studies have been conducted on its role in microglial and astrocytic inflammations.

293-296: Overall, comparing the results of our research with other researchers regarding the relationship between the intensity of inflammation and Nurr1 expression, the results of most studies are consistent with our results and prove that MGCs under LPS or Aβ stress show lower levels of Nurr1 expression [29, 48].
